# ^68^Ga-PSMA-HBED-CC PET/CT imaging for adenoid cystic carcinoma and salivary duct carcinoma: a phase 2 imaging study

**DOI:** 10.7150/thno.38501

**Published:** 2020-01-12

**Authors:** Wim van Boxtel, Susanne Lütje, Ilse C.H. van Engen-van Grunsven, Gerald W. Verhaegh, Jack A. Schalken, Marianne A. Jonker, James Nagarajah, Martin Gotthardt, Carla M.L. van Herpen

**Affiliations:** 1 Department of Medical Oncology, Radboud university medical center, Nijmegen, the Netherlands; 2 Department of Radiology and Nuclear Medicine, Radboud university medical center, Nijmegen, the Netherlands; 3 Department of Nuclear Medicine, University Hospital Bonn, Bonn, Germany; 4 Department of Pathology, Radboud university medical center, Nijmegen, the Netherlands; 5 Department of Urology, Radboud university medical center, Nijmegen, the Netherlands; 6 Department of Health Evidence, Radboud university medical center, Nijmegen, the Netherlands; 7 Department of Nuclear Medicine, Technical University Munich, Germany

**Keywords:** Prostate-specific membrane antigen, Positron-Emission Tomography, Immunohistochemistry, Adenoid cystic carcinoma, Salivary duct carcinoma

## Abstract

**Rationale**: Treatment options for recurrent and/or metastatic (R/M) adenoid cystic carcinoma (ACC) and salivary duct carcinoma (SDC), major subtypes of salivary gland cancer, are limited. Both tumors often show overexpression of prostate-specific membrane antigen (PSMA). In prostate cancer, PSMA-ligands labeled with ^68^Ga or ^177^Lu are used for imaging and therapy, respectively. Primary aim of this study in R/M ACC and SDC patients was to systematically investigate ^68^Ga-PSMA-uptake by PET/CT imaging to determine if PSMA radionuclide therapy could be a treatment option.

**Methods**: In a prospective phase II study, PET/CT imaging was performed 1 h post injection of ^68^Ga-PSMA-HBED-CC in 15 ACC patients and 10 SDC patients. Maximum standardized uptake values (SUV) were determined in tumor lesions. Immunohistochemical PSMA expression was scored in primary tumors and metastatic tissue. Standard imaging (MRI or CT) was performed for comparison.

**Results**: In ACC patients, SUV_max_ ranged from 1.1 to 30.2 with a tumor/liver-ratio >1 in 13 out of 14 evaluable patients (93%). In SDC patients, SUV_max_ ranged from 0.3 to 25.9 with a tumor/liver-ratio >1 in 4 out of 10 patients (40%). We found a large intra-patient inter-metastatic variation in uptake of ^68^Ga-PSMA, and immunohistochemistry did not predict ligand uptake in ACC and SDC. Finally, PSMA-PET detected additional bone metastases compared to CT in 2 ACC patients with unexplained pain.

**Conclusion**: In 93% of ACC patients and 40% of SDC patients we detected relevant PSMA-ligand uptake, which warrants to study PSMA radionuclide therapy in these patients. Additionally, our data provide arguments for patient selection and treatment timing. Finally, PSMA-PET imaging has added diagnostic value compared to CT in selected patients.

## Introduction

Salivary gland cancer (SGC) is a rare cancer with an annual incidence rate of 0.4-2.6 cases per 100,000 people 1. SGCs are histologically diverse with 22 different subtypes 1. Adenoid cystic carcinoma (ACC) and salivary duct carcinoma (SDC) are major subtypes in which recurrent and metastatic (R/M) disease is common. The survival of R/M ACC can be many years and metastases are frequently slow growing. Palliative systemic treatment is offered in case of symptomatic and/or rapid progressive disease. First-line options include single-agent vinorelbine or mitoxantrone, or cyclophosphamide plus doxorubicin plus cisplatin (CAP). Evidence for second-line therapy is lacking 2. SDC is one of the most aggressive SGCs. In R/M disease the median overall survival with best supportive care is only 5 months 3. Therefore, palliative systemic treatment should not be delayed. Androgen deprivation therapy (ADT) is often given as first-line treatment, as the majority of SDCs are androgen receptor (AR)-positive 4, 5. Because 21-44% of SDCs have *ERBB2* (encoding HER2) amplification, a subset of patients can also be treated with HER2-targeted therapies 6, 7. Despite these treatment options, survival in R/M SDC patients is limited. Therefore, in both ACC and SDC new treatment strategies are urgently needed.

Prostate-specific membrane antigen (PSMA) is a membrane glycoprotein first detected on the human prostatic carcinoma cell line LNCaP 8. Subsequently, expression has been shown in the neovasculature of a wide variety of tumors 9. Highly specific ligands for PSMA have been developed which can be labeled with radioisotopes such as Gallium-68 (^68^Ga) or Fluor-18 (^18^F) for imaging. In prostate cancer, ^68^Ga-PSMA-PET/CT imaging visualized substantially more tumor lesions than reported for other imaging modalities, such as CT, ^18^F-FDG-PET, ^11^C-choline-PET and MRI 10. Moreover, labeling PSMA-ligands with β-emitting radionuclides such as Lutetium-177 (^177^Lu) or α-emitting radionuclides such as Actinium-225 (^225^Ac) are promising options for radionuclide therapy. Results of ^177^Lu-PSMA radionuclide therapy in metastatic castration resistant prostate cancer (mCRPC) patients who failed conventional therapeutic options showed a prostate-specific antigen decrease in 56 - 80.4% of patients, a favorable safety profile, and a median progression free survival of 13.7 months in retrospective studies 11-13. A prospective phase 2 study in mCRPC patients established a radiological response rate of 82% in 17 evaluable patients 14 and currently, a phase 3 trial on ^177^Lu-PSMA in mCRPC is recruiting (VISION trial, NCT03511664).

Evaluation of the normal biodistribution of ^68^Ga-PSMA in prostate cancer patients revealed high uptake in the salivary glands 15. Furthermore, we previously demonstrated high PSMA-ligand uptake in a patient with R/M ACC using ^68^Ga-PSMA-PET/CT 16. Subsequently, Klein Nulent et al. described ^68^Ga-PSMA-PET/CT in 9 patients with R/M ACC in a retrospective case series 17. All patients showed PSMA-ligand uptake in local recurrences and distant metastases. In SDC, PSMA-PET imaging has not been evaluated, yet.

In this prospective phase 2 study we investigated ^68^Ga-PSMA-ligand uptake using PSMA-PET/CT imaging in R/M ACC and SDC patients. Our secondary goals were to correlate ligand uptake to immunohistochemical (IHC) PSMA-expression, to establish the diagnostic added value of ^68^Ga-PSMA-PET imaging over the current standard, to investigate the normal biodistribution of ^68^Ga-PSMA in ACC and SDC patients, and to investigate the difference in PSMA-ligand uptake between ADT-treated and ADT naïve SDC patients. These data may provide a rationale for PSMA radionuclide therapy in R/M ACC and SDC patients with relevant PSMA-ligand uptake in tumor lesions.

## Methods

### Study population

Patients with R/M ACC or SDC who were ≥ 18 years old and able to provide a written informed consent were recruited from the Radboud university medical center, a tertiary referral hospital specialized in salivary gland cancer in the Netherlands. Contra-indications to participate were a contra-indication for PET imaging (pregnancy, breast feeding, severe claustrophobia), impaired renal function (MDRD <30 ml/min/1.73 m^2^), and impaired liver function (AST and ALT ≥ 2.5 x upper limit of normal (ULN) or ≥ 5 x ULN for patients with liver metastases). All study procedures were in accordance to the declaration of Helsinki. The study protocol was approved by the local medical ethics committee (file code 2017-3455) and the study protocol was published on www.clinicaltrials.gov (Identifier: NCT03319641).

Standard imaging consisted of contrast-enhanced full-dose CT of the neck, chest and/or abdomen (depending on disease localization) after an intravenous injection of 100 mL of an iodine-based contrast agent (Ultravist; Bayer Schering Pharma, Berlin, Germany) performed on a Biograph mCT (Siemens Healthcare, Erlangen, Germany). All patients with a local recurrence or known intracerebral disease underwent an MRI scan of the head and neck after intravenous injection of 7.5 ml gadobutrol (1mmol/ml). Imaging was performed on a 3 T unit (Achieva 3TX, Philips Medical Systems, Best, The Netherlands). All MRI and CT scans were performed within 4 weeks before or after the PSMA-PET scan.

### ^68^Ga-PSMA-HBED-CC PET/CT imaging

Gallium-68 is a short-lived radionuclide (T_1/2_ 68 minutes) decaying 89% through positron emission (maximum energy of 1.92 MeV, mean 0.89 MeV) 18. Conjugation of ^68^Ga with the HBED-CC chelator of the PSMA-specific pharmacophore Glu-NHCO-NH-Lys was performed as described previously 19 and obtained from ABX (Dresden, Germany). Radiolabeling and purification of the PSMA-ligand were performed with an automated module. Typically, the labeling efficiency exceeded 98% as determined by individual quality control of each synthesis. All patients received intravenous administration of 3 MBq/kg of the ^68^Ga-PSMA ligand. All preparations contained 10 μg of the PSMA ligand. Patients were scanned from skull to proximal femora approximately at 1 h post injection and were asked to empty their bladder before tracer injection and before PET-scan. The acquisition time for each of up to 7 bed positions (16.2 cm; overlapping scale, 4.2 cm) was 2 minutes with a 15.5 cm step of the bed. Random, scatter, and decay correction was applied for all emission data. Reconstructions were conducted with an ordered subset expectation maximization algorithm with 3 iterations/21 subsets and Gauss filtered to a transaxial resolution of 4 mm at full-width at half-maximum. Attenuation correction was performed using a low-dose CT (presets 40mAs and 120 kV). The qualitative analysis of ^68^Ga-PSMA-HBED-CC PET/CT scans was performed lesion based and in comparison to acquired full-dose CT and MRI scans. The images acquired by the PET component of the PET/CT system were initially categorized upon the presence of tumor lesions based on visual evaluation and, if present, into soft-tissue lesions originating from the salivary gland or salivary gland bed, other soft-tissue lesions, lymph node lesions, or distant metastatic lesions. Data analysis was performed using Oasis software (Segami Corporation, Columbia, Maryland, USA). For lung metastases, only metastases of ≥1 cm were evaluated because of the partial volume effect in smaller metastases and motion artifacts.

Quantitative analysis of ^68^Ga-PSMA-HBED-CC ligand uptake was performed by measuring SUV_max_ in tumors and metastases as it is standard in clinical routine. Metastases were identified on the CT-scan first, allowing measurement of metastases, which did not exceed background ligand uptake. In addition, SUV_max_ and SUV_mean_ (region of interest 2.4 cm in diameter) were measured in the lacrimal gland, major salivary glands (unaffected parotid, submandibular and sublingual gland), lung, liver, duodenum, spleen, kidney, bladder, gluteus muscle and mediastinal bloodpool in order to assess the normal biodistribution. Tumor/parotid, tumor/liver, and tumor/muscle ratios were calculated by dividing SUV_max_ in the tumor by SUV_mean_ in the reference organs. A tumor/liver ratio of >1 was regarded as relevant PSMA-ligand uptake 20.

### Immunohistochemistry

Formalin-fixed, paraffin-embedded (FFPE) tissue blocks of resected or biopsied primary tumors and metastases were requested and 4 μm sections were cut. Sections were stained immunohistochemically using fully automated protocols on the Benchmark XT (Ventana Medical Systems, Tucson, AZ, USA) using the mouse antihuman PSMA monoclonal antibody (3E6; DAKO, Carpinteria, CA) directed against the internal domain of the PSMA as described before 17. PSMA stained slides were scored for percentage of positive tumor cells and presence of PSMA-staining in the neovasculature of tumors.

### Statistical analysis

Primary aim of this study was to assess PSMA-ligand uptake in R/M ACC and SDC patients in order to establish eligibility for a future study on PSMA radionuclide therapy. PSMA-ligand uptake was described per disease site (mean and standard deviation in e.g. lung and liver metastases) in every patient in order to show inter-metastatic and inter-site variation. Analyses on secondary outcome measures were performed to summarize data and to support hypotheses about rational patient selection for a future PSMA radionuclide therapy study, but are only hypothesis generating. Scatter-plots were made to show the variation in PSMA-ligand uptake, correlation between time from diagnosis/recurrence to PSMA-PET scan and PSMA-ligand uptake, and the correlation between IHC PSMA expression and PSMA-ligand uptake. Analyses were performed using SPSS version 25.0.

## Results

### Patient characteristics

Fifteen patients with R/M ACC (11 men, 4 women) and 10 patients with R/M SDC (all men) were included between November 20^th^, 2017 and May 14^th^, 2018. The ACC patients had a median age of 58 years (range 44-76 years). ACC originated from the salivary glands in 12 patients, and from the trachea, the bronchus and the Bartholin gland in the other 3 patients. Local recurrences, regional recurrences, and distant metastases were present in 3, 2, and 14 out of 15 ACC patients, respectively. SDC patients had a median age of 69.5 years (range 55-79 years). Local recurrences, regional recurrences, and distant metastases were present in 3, 4, and 9 out of 10 SDC patients, respectively. Further patient characteristics are listed in Supplementary Table 1. Normal biodistribution of ^68^Ga-PSMA in ACC and SDC patients is listed in Supplementary Table 2. In the ACC patients, no statistical differences in the normal biodistribution between men and women were found.

### PSMA-ligand uptake in tumors and metastases

**Figure 1:**
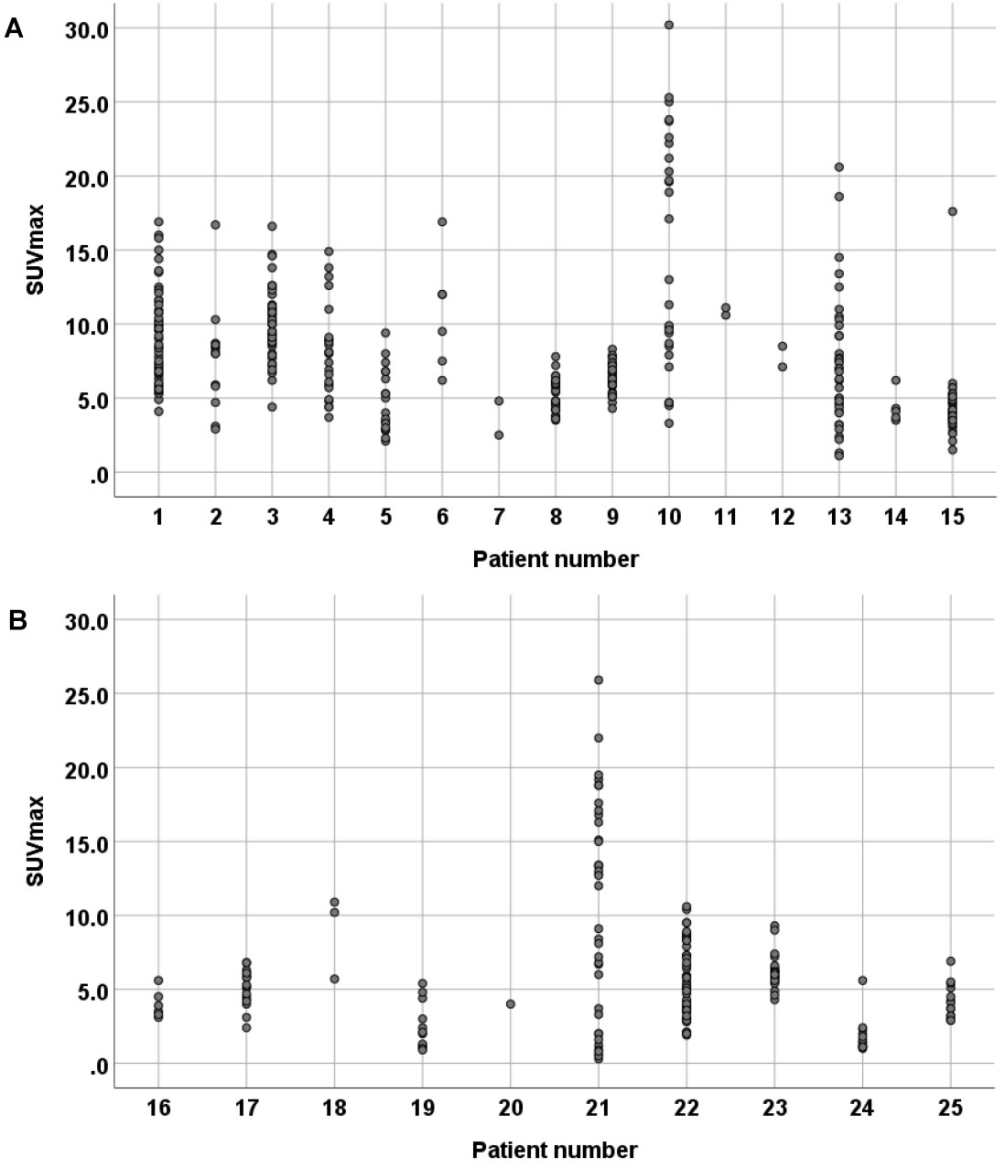
Scatter-plot of intra-patient inter-metastatic variation in maximum standardized uptake value's (SUVmax) in adenoid cystic carcinoma patients (**A**) and salivary duct carcinoma (SDC) patients (B). The female ACC patients (patient 2, 5, 7, 12) had a PSMA-ligand uptake of 6.13 ± 2.99, compared to 8.16 ± 4.51 in the male ACC patients. The SDC patients receiving ADT (patient 16, 19, 20, 23, 25) had a PSMA-ligand uptake of 4.59 ± 1.91, compared to 6.65 ± 5.14 in the SDC patients not receiving ADT (patient 17, 18, 21, 22, 24).

**Figure 2:**
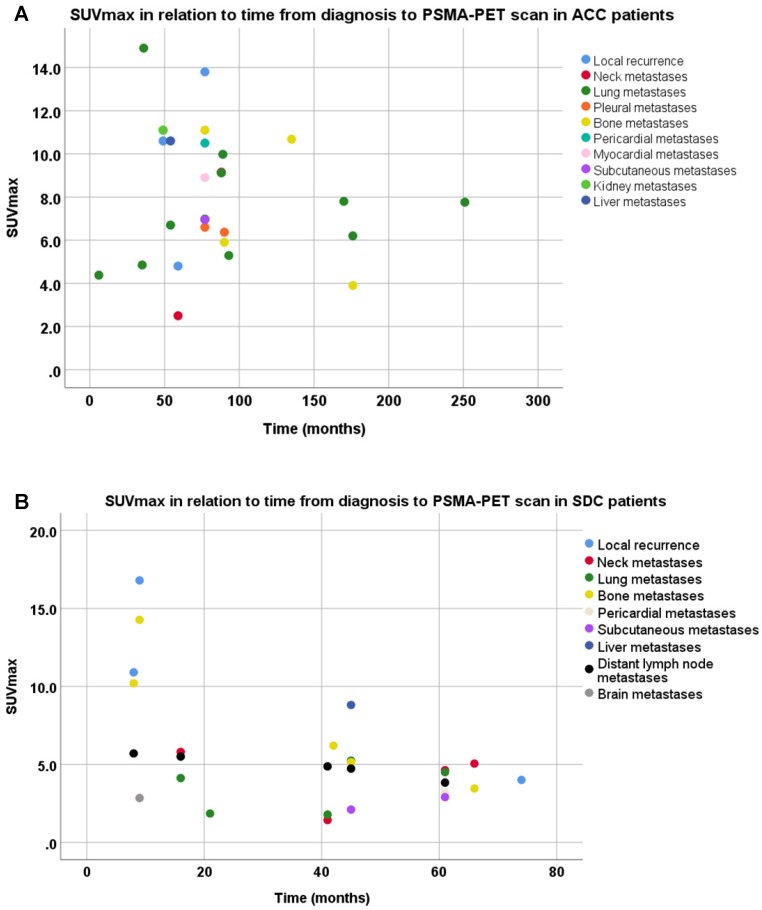
Scatter plot of the time from diagnosis to PSMA-PET scan and maximum standardized uptake value's (SUV_max_) per disease site in recurrent and metastatic lesions of adenoid cystic carcinoma patients (**A**) and salivary duct carcinoma patients (**B**). Note that patients with x disease site are represented in the figures x times.

**Figure 3:**
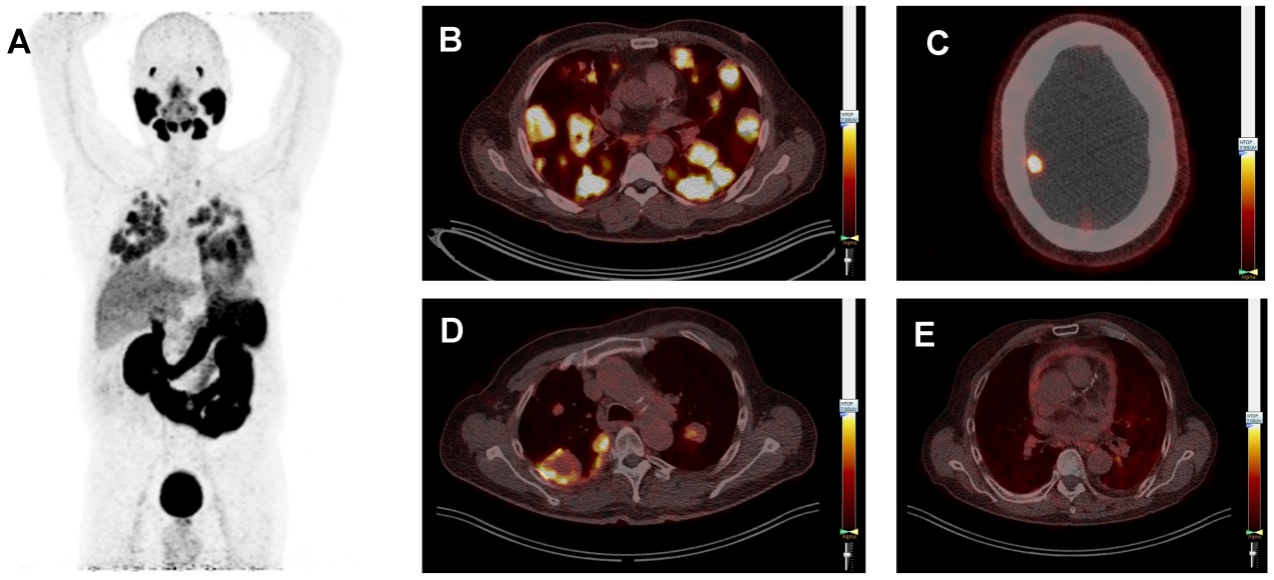
Typical prostate-specific membrane antigen (PSMA) positron-emission tomography (PET) whole body image of patient 3 (ACC) showing PSMA-ligand uptake in the lacrimal glands, salivary glands, lung metastases, liver, kidneys, spleen, intestine and bladder (**A**). Typical PSMA-PET/CT fusion images, all scaled at a SUV_max_ of 7.0, showing multiple lung metastases with homogeneous PSMA-ligand uptake in the same patient (**B**). Patient 21 (SDC) showing a brain metastasis (**C**). Patient 13 (ACC) showing lung metastases with inhomogeneous PSMA-ligand uptake and central photopenia. This patient was treated with cyclophosphamide plus doxorubicin plus cisplatin at the time of the PSMA-PET scan (**D**), and patient 25 (SDC) showing pericarditis carcinomatosa (**E**).

SUV_max_ were determined in all recurrent tumors and metastatic lesions (Table 1). In ACC patients, we found SUV_max_ ranging from 1.1 to 30.2 and a tumor/liver ratio of >1 in all tumor sites in 13 out of 14 evaluable ACC patients (93%). In SDC patients, we found SUV_max_ ranging from 0.3 to 25.9 and a tumor/liver ratio >1 in all tumor sites in 4 out of 10 SDC patients (40%). Moreover, a large intra-patient inter-metastatic variation in PSMA uptake was found in ACC as well as SDC patients (Figure 1). In ACC patients, PSMA-ligand uptake was 6.13 ± 2.99 in female patients, compared to 8.16 ± 4.51 in male patients. In SDC patients, a negative trend was found between PSMA-ligand uptake and time from primary diagnosis to PSMA-PET scan, as shown in Figure 2. The same was true for PSMA-ligand uptake and time from recurrence to PSMA-PET scan, as shown in Supplementary Figure 1. In ACC patients, no correlations between time from diagnosis/recurrence to PSMA-PET scan and PSMA-ligand uptake were seen. Exemplary PSMA-PET images are shown in Figure 3.

### Influence of treatment on PSMA-ligand uptake

Of the 15 ACC patients, 12 patients received no systemic therapy at the time of the PSMA-PET scan, 1 patient received vorinostat, 1 patient received cabozantinib, and 1 patient received CAP once every 3 weeks. Of the 10 SDC patients, 3 patients received no systemic therapy, 5 patients received ADT, 1 patient received trastuzumab plus pertuzumab, and 1 patient received trastuzumab-emtansine. The ACC patient receiving CAP (patient 13) showed a pronounced inhomogeneous uptake with central photopenia in lung and liver metastases, as shown in Figure 3c.The SDC patients receiving ADT had a PSMA-ligand uptake of 4.59 ± 1.91 (mean ± SD), compared to 6.65 ± 5.14 in the patients not receiving ADT. However, time from diagnosis was longer for patients receiving ADT than for patients not receiving ADT (56.8 ± 14.7 months vs. 19.8 ± 15.1 months). The other patients did not show remarkable differences in PSMA-ligand uptake.

### Immunohistochemistry

**Figure 4:**
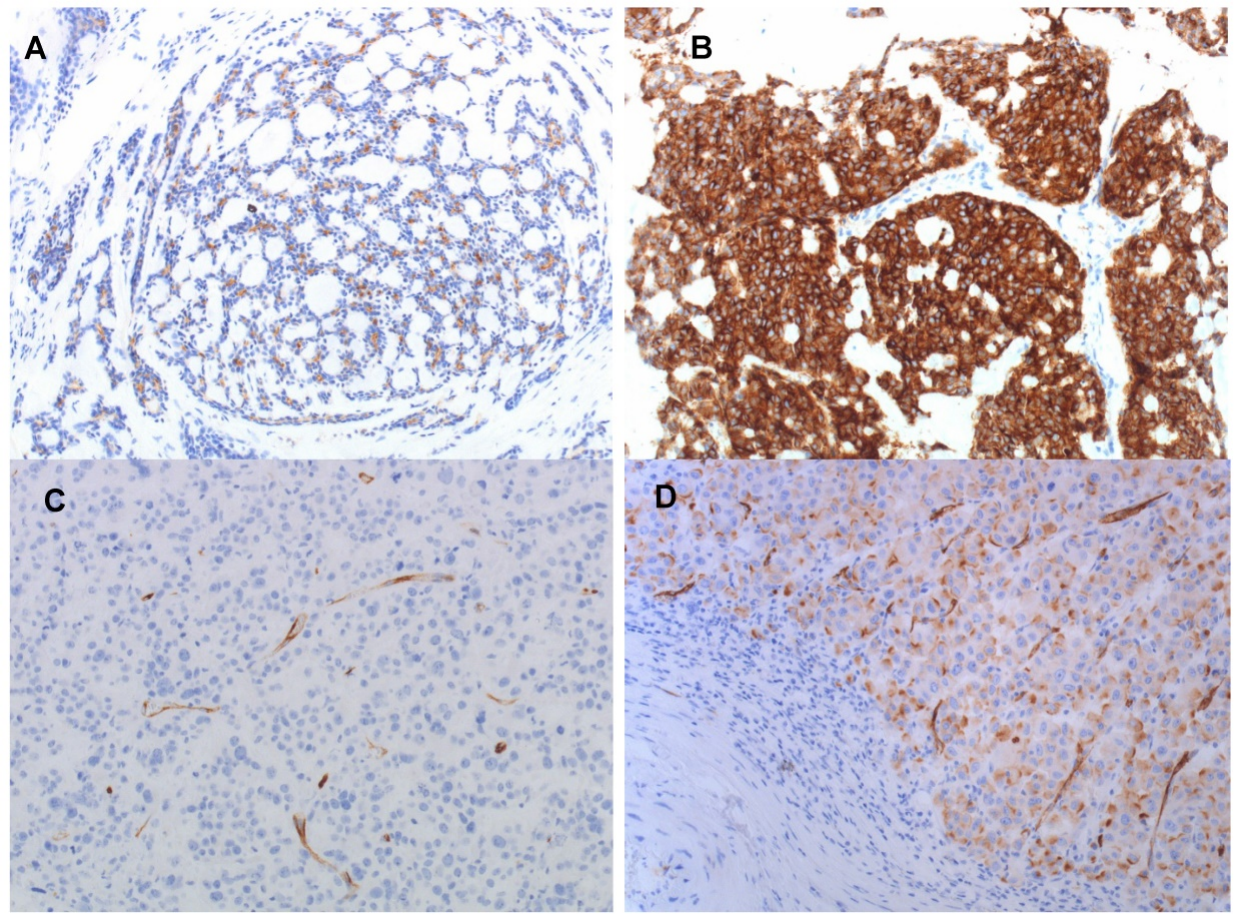
Immunohistochemical PSMA expression in primary tumors of ACC patients no. 4 (**A**), no. 10 (**B**) and SDC patients no. 17 (**C**) and no. 21 (**D**). PSMA expression in tumor cells is 10% in (**A**), 90% in (**B**), negative in (**C**), and 50% in (**D**). PSMA expression in tumor associated neovasculature is negative in the ACC patients (**A** and **B**), and positive in most SDC patients, as shown in (**C** and **D**). Magnification 200x.

In the ACC patients, the median percentage of PSMA-positive tumor cells was 7.5% (range 0-90%) in the resected primary tumors and 5% (range 0-80%) in 11 biopsies from metastases. IHC PSMA expression in the tumor-associated neovasculature was negative in all ACC patients. In SDC patients, median percentage of PSMA-positive tumor cells was 0% (range 0-50%) with 5 out 9 patients showing no PSMA-expression in the resected primary tumors, and 0% in one biopsied metastatic lesion. IHC PSMA expression in the tumor-associated neovasculature was positive in 8 of 9 evaluable SDC patients. See Figure 4 for exemplary IHC PSMA-expression patterns. Furthermore, Supplementary Figures 2a and 2c show scatter plots of the IHC PSMA expression in the resected primary tumor and mean SUV_max_ in R/M disease sites in ACC and SDC patients, respectively. Supplementary Figure 2b shows a scatter plot of the IHC PSMA expression in biopsied R/M lesions and mean SUV_max_ in the same disease site in ACC patients.

### Diagnostic added value

**Figure 5:**
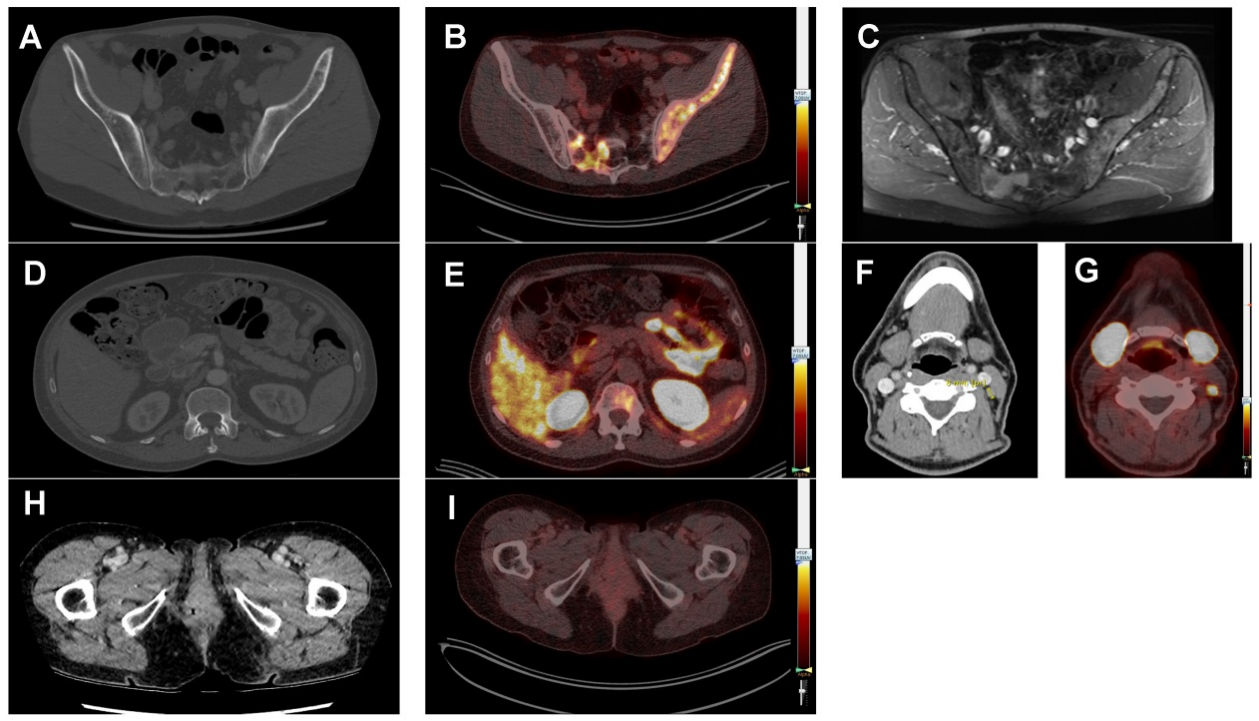
Diagnostic added value of prostate-specific membrane antigen (PSMA) positron-emission tomography (PET) imaging compared to standard imaging. All PSMA-PET fusion images are scaled at a SUV_max_ of 7.0. Patient 4 (ACC) suffered from pain in his left hip region. The full-dose CT-scan did not show bone metastases (**A**), but the concurrent PSMA-PET scan did (**B**). Subsequently, an MRI scan was performed for comparison, which also showed the bone metastases (**C**). Patient 9 (ACC) suffered from back pain. The full-dose CT-scan did not show a bone metastasis (**D**), but the PSMA-PET scan did (**E**). patient 1 (ACC) showed a borderline enlarged lymph node on the full-dose CT-scan (**F**). On the PSMA-PET scan this node was PSMA-avid with a SUV_max_ of 9.9 (**G**) and therefore suspected to be a neck metastasis. This was not histologically proven because of lack of consequences. In patient 2, a local recurrence of an ACC of the Bartholin gland was suspected based on the full-dose CT-scan (**H**). However, this patient had high PSMA-ligand uptake in the lung metastases, but was negative for local recurrence (**I**). Evaluation by a gynecologic oncologist revealed indeed no recurrence, but scar tissue.

PSMA-PET imaging had added diagnostic value in 4 ACC patients (Figure 5): Two ACC patients suffered from back and hip pain without abnormalities on the full-dose CT scan, but the PSMA-PET scan revealed bone metastases in the thoracic spine and hip, respectively. In one ACC patient additional lymph node metastases were observed without therapeutic consequences. Finally, in one patient local recurrence of ACC of the Bartholin gland was suspected based on the full-dose CT-scan. However, this patient had high PSMA-ligand uptake in her lung metastases, but the site of the primary tumor was negative. Evaluation by a gynecologic oncologist revealed indeed no recurrence, but scar tissue in the Bartholin gland.

## Discussion

Here we describe the first prospective study on ^68^Ga-PSMA-PET imaging in R/M ACC and SDC patients. We found relevant PSMA-ligand uptake in 93% of ACC patients and 40% of SDC patients with a tumor/liver ratio >1. In mCRPC, eligibility for ^177^Lu-PSMA radionuclide therapy is based on a positive ^68^Ga-PSMA-PET scan defined as tumor/liver ratio >1, as is the case in the currently recruiting phase 3 trial (VISION, NCT03511664). Based on the results from this study, we propose a prospective phase II study with ^177^Lu-PSMA radionuclide therapy in R/M ACC and SDC patients.

In order to optimize efficacy of PSMA radionuclide therapy in such a study, the following factors are of importance. First, we found a large intra-patient inter-metastatic variation in PSMA uptake, which can influence treatment response. Second, we found a negative trend between PSMA-ligand uptake and time from diagnosis to PSMA-PET scan in SDC patients, which could be an argument for PSMA radionuclide therapy early in disease. This is in contrast with prostate cancer in which PSMA expression increases with increasing grade, stage, and evolution to castration resistance 21. Third, the decision to treat patients with the β-emitting radionuclide ^177^Lu or the α-emitting radionuclide ^225^Ac may be vital. Alpha-emitting radionuclides have a higher linear energy transfer and a shorter path length, resulting in higher doses to tumors coupled with a greater probability of generating double-strand DNA breaks. However, due to the higher radiation range of ^177^Lu compared to ^225^Ac, uptake in the neovasculature may be sufficient to deliver tumoricidal radiation doses. Therefore, we believe that ^177^Lu may be preferable for treating patients who do not show immunohistochemical PSMA expression in the tumor cells but show sufficient ^68^Ga-PSMA ligand uptake. Furthermore, α-emitting radionuclides induce xerostomia more often, which is already the major dose-limiting toxicity in CRPC patients 22. This may be an even more important issue in salivary gland cancer patients who had a salivary gland resection and postoperative radiotherapy in most cases.

Safety is another point of concern for a future study on PSMA radionuclide therapy. Data from prostate cancer indicate that a higher tumor load is beneficial, as this results in a lower uptake in dose-limiting organs (the so called “sink-effect”) 23, 24. On the other hand, ACC patients can develop large and numerous lung metastases, and radionuclide therapy may induce radiation fibrosis in the surrounding lung tissue, inducing respiratory failure. This could be an argument for PSMA radionuclide therapy earlier in the course of disease. Furthermore, in mCRPC patients treated with ^177^Lu-PSMA, the presence of visceral metastases is a negative predictive factor for OS, providing an argument for reconsidering treatment sequencing in these patients 25. Moreover, disease localization in ACC and SDC is different compared to prostate cancer, as SDC patients can develop brain metastases and ACC patients can develop local recurrences with intracerebral growth. Because mCRPC patients rarely develop intracerebral disease, the effects of radionuclide therapy on the brain is largely unknown, although some data exist 26, 27. Finally, dosimetry analyses should be done to quantify the applied target doses. Uptake and retention are the two pillars of radioligand therapies in order to deliver significant doses to target lesions. In this study, we performed only a single time PET scan, which mainly mirrors the uptake but provides no insight to the retention ability of the tumors. However, we anticipate that a relevant uptake itself will already deliver tumoricidal doses to the target lesion as already shown. We advocate to include a dosimetry approach in future studies to quantify the doses delivered to the tumors.

IHC PSMA expression of the primary tumor did not reliably predict PSMA-ligand uptake in ACC and SDC patients, as some patients without IHC PSMA expression showed good PSMA-ligand uptake. Therefore, IHC PSMA expression cannot be used in clinical practice to select patients for ^68^Ga-PSMA-PET imaging. In a retrospective analysis on PSMA-PET in 9 ACC patients, the published raw data also showed discrepancies between IHC PSMA expression and PSMA-ligand uptake 17. In that study, all patients showed IHC PSMA expression, but some patients with low IHC PSMA expression showed intense PSMA-ligand uptake, and others with high IHC PSMA expression showed no PSMA-ligand uptake. These results suggest that accumulation of PSMA-ligands in the salivary glands is non-specific, or at least not correlated with IHC PSMA expression. This is in line with other studies, for instance Rupp et al. who showed that the high accumulation of PSMA radioligands in salivary glands does not correspond to high PSMA expression levels determined by autoradiography and IHC, indicating a mainly non-PSMA related radioligand uptake in salivary glands 28. This is in contrast to data in prostate cancer, in which a strong correlation was found between IHC PSMA expression and PSMA ligand uptake 29, 30. Furthermore, it is interesting to note that IHC PSMA expression in normal salivary glands is limited to the intercalated ducts 28. Because ACC is a tumor arising from the intercalated duct cells, and SDC a tumor arising from excretory duct cells 31, PSMA-ligand uptake may be more intense and more PSMA-specific in ACC compared to SDC.

In this study, SDC patients receiving ADT during the PSMA-PET scan had a lower PSMA-ligand uptake than SDC patients not receiving ADT, but time from diagnosis to PSMA-PET scan was also longer for ADT-treated patients. The lower PSMA-ligand uptake in ADT-treated patients is remarkable because data from prostate cancer patients showed a higher PSMA-ligand uptake after ADT 32. Therefore, it would be of interest to evaluate PSMA-ligand uptake before and after ADT in SDC patients in order to establish the difference in individual patients. Because women have physiologically lower levels of androgens, we evaluated the differences in PSMA-ligand uptake between the female and male ACC patients. However, PSMA-ligand uptake was higher in men, but the groups were too small for a reliable statistical analysis (4 women vs. 11 men).PSMA-PET imaging has added diagnostic value in part of the ACC patients compared to standard imaging. In our series, two ACC patients had a false negative full-dose CT and one patient had a false positive full-dose CT whereas PSMA-PET imaging was correctly positive and negative, respectively. Therefore, we recommend adding a PSMA-PET scan to standard imaging in ACC patients in case of discrepancy between the clinical symptoms and standard imaging results. However, it does not seem appropriate to use PSMA-PET routinely based on the current evidence, because of lack of added diagnostic value in most patients and costs.

## Conclusion

PSMA-PET imaging showed relevant PSMA-ligand uptake in local recurrences and metastases in 93% of ACC patients and 40% of SDC patients. Therefore, a phase II study with PSMA radionuclide therapy in these patients is warranted. Variation in PSMA-ligand uptake, tumor volume, location of metastases, as well as timing of radionuclide therapy may influence efficacy and toxicity. Furthermore, PSMA-PET imaging may have added diagnostic value compared to standard imaging, especially in case of unexplained pain.

## Supplementary Material

Supplementary figures and tables.

## Figures and Tables

**Table 1:** Maximum standardized uptake value's (SUV_max_) and immunohistochemical (IHC) prostate-specific membrane antigen staining in adenoid cystic carcinoma patients (No. 1-15) and salivary duct carcinoma patients (No 16-25). SD: standard deviation. ^#^ Immunohistochemical PSMA expression was assessed in available tissue of primary tumors and biopsied metastases. * ratios could not be calculated because the scan in patient no. 6 was terminated prematurely because the patient could not lay still.

No.	Tumor site (number of lesions)	Mean SUV_max_ (SD)	Homogeneous?	Tumor/organ ratios					Immunohistochemistry^#^	
				Tumor/parotid	Tumor/liver	Tumor/muscle	Tumor/kidney	Tumor/blood	Tumor (%)	Neovasculature
**Adenoid cystic carcinoma patients**										
1	Resected primary tumor								<5%	Neg
	Neck metastases (2)	9.15 (1.06)	Yes	0.28	1.48	13.1	0.12	4.36	-	-
	Lung metastases (53)	9.12 (3.22)	Yes	0.28	1.47	13.0	0.12	4.34	<5%	Neg
2	Resected primary tumor								0%	Neg
	Lung metastases (16)	7.76 (3.29)	No	0.50	1.49	19.4	0.31	7.05	0%	Neg
3	Resected primary tumor								30%	Neg
	Lung metastases (51)	9.98 (2.26)	Yes	0.52	2.50	25.0	0.39	9.07	5%	Neg
4	Resected primary tumor								10%	Neg
	Local recurrence (1)	13.80	Yes	0.71	3.83	46.0	0.36	17.25	-	-
	Lung metastases (10)	6.98 (2.68)	Yes	0.36	1.94	23.3	0.18	8.73	-	-
	Pleural metastasis (1)	6.60	No	0.34	1.83	22.0	0.17	8.25	-	-
	Pericardial metastases (2)	10.5 (6.22)	Yes	0.54	2.92	35.0	0.27	13.13	-	-
	Myocardial metastasis (1)	8.90	Yes	0.46	2.47	29.7	0.23	11.13	-	-
	Subcutaneous metastases (6)	6.95 (1.84)	Yes	0.36	1.93	23.2	0.18	8.69	20%	Neg
	Bone metastases (3)	11.1 (2.05)	Yes	0.57	3.08	37.0	0.29	13.87	10%	Neg
5	Resected primary tumor								-	-
	Lung metastases (18)	4.85 (2.13)	Yes	0.26	1.18	16.2	0.14	5.39	-	-
6	Resected primary tumor								10%	Neg
	Bone metastases (6)	10.68 (3.84)	Yes	*	*	26.7	*	*	-	-
7	Resected primary tumor								2%	Neg
	Local recurrence (1)	4.8	Yes	0.32	1.17	12.0	0.15	4.00	-	-
	Neck metastasis (1)	2.5	Yes	0.16	0.61	6.3	0.08	2.08	-	-
8	Resected primary tumor								0%	Neg
	Lung metastases (28)	5.29 (1.12)	No	0.60	1.60	10.6	0.24	4.41	-	-
9	Resected primary tumor								30% weak	Neg
	Pleural metastases (29)	6.37 (1.05)	No	0.37	1.42	21.2	0.18	5.79	0%	Neg
	Bone metastasis (1)	5.9	No	0.34	1.31	19.7	0.17	5.36	-	-
10	Resected primary tumor								90%	Neg
	Lung metastases (27)	14.90 (7.89)	Yes	1.04	3.73	37.3	0.36	11.46	80%	Neg
11	Resected primary tumor								10%	Neg
	Local recurrence (1)	10.6	Yes	0.62	2.86	35.3	0.23	8.15	-	-
	Kidney metastasis (1)	11.1	Yes	0.65	3.00	37.0	0.24	8.54	-	-
12	Resected primary tumor								<1%	Neg
	Lung metastases (2)	7.80 (0.99)	No	0.80	1.90	39.0	0.21	9.75	<5%	Neg
13	Resected primary tumor								10%	Neg
	Lung metastases (30)	6.70 (4.57)	No	0.86	2.68	16.8	0.13	6.70	5%	Neg
	Liver metastases (6)	10.60 (1.99)	No	1.36	4.24	26.5	0.21	10.60	5%	Neg
14	Resected primary tumor								0%	Neg
	Lung metastasis (1)	6.20	Yes	0.35	1.77	12.4	0.26	5.17	-	-
	Bone metastases (4)	3.90 (0.37)	Yes	0.22	1.11	7.8	0.16	3.25	-	-
15	Resected primary tumor								5%	Neg
	Lung metastases (43)	4.38 (2.28)	No	0.35	1.37	11.0	0.16	4.87	0%	Neg
**Salivary duct carcinoma patients**										
16	Resected primary tumor								0%	Pos
	Neck metastases (2)	5.05 (0.78)	Yes	0.30	0.84	12.6	0.14	3.61	-	-
	Bone metastases (4)	3.45 (0.34)	Yes	0.20	0.58	8.6	0.09	2.46	0%	Pos
17	Resected primary tumor								0%	Pos
	Neck metastasis (1)	5.8	No	0.39	1.87	14.5	0.09	5.27	-	-
	Lung metastases (5)	4.12 (1.68)	No	0.28	1.33	10.3	0.07	3.75	-	-
	Distant lymph nodes (12)	5.50 (0.79)	No	0.37	1.77	13.8	0.09	5.00	-	-
18	Resected primary tumor								0%	Pos
	Local recurrence (1)	10.9	Yes	0.70	2.66	27.3	0.31	9.08	-	-
	Distant lymph node (1)	5.7	Yes	0.37	1.39	14.3	0.16	4.75	-	-
	Bone metastasis (1)	10.2	Yes	0.65	2.49	25.5	0.29	8.50	-	-
19	Resected primary tumor								<1%	Pos
	Neck metastases (3)	1.43 (0.49)	No	0.10	0.45	4.8	0.03	1.30	-	-
	Lung metastases (6)	1.78 (0.85)	No	0.12	0.56	5.9	0.04	1.62	-	-
	Distant lymph nodes (3)	4.87 (0.50)	No	0.34	1.52	16.2	0.11	4.43	-	-
20	Resected primary tumor								<5%	Neg
	Local recurrence (1)	4.0	Yes	0.23	2.00	13.3	0.34	4.44	-	-
21	Resected primary tumor								50%	Pos
	Local recurrence (1)	16.8	Yes	1.19	2.63	56.0	0.52	14.00	-	-
	Brain metastases (13)	2.84 (3.60)	Yes	0.20	0.44	9.5	0.09	2.37	-	-
	Bone metastases (23)	14.27 (5.22)	Yes	1.01	2.23	47.6	0.44	11.89	-	-
22	Resected primary tumor								0%	Pos
	Lung metastases (28)	5.24 (2.22)	No	0.46	1.34	10.5	0.23	3.49	-	-
	Distant lymph nodes (4)	4.73 (0.52)	Yes	0.41	1.21	9.5	0.20	3.15	-	-
	Liver metastases (7)	8.81 (1.25)	Yes	0.77	2.26	17.6	0.38	5.87	-	-
	Bone metastases (8)	5.14 (2.20)	No	0.45	1.32	10.3	0.22	3.43	-	-
	Subcutaneous metastases (2)	2.10 (0.00)	Yes	0.18	0.54	4.2	0.09	1.40	-	-
23	Resected primary tumor								-	-
	Bone metastases (22)	6.20 (1.20)	Yes	0.38	1.72	20.7	0.19	5.17	-	-
24	Resected primary tumor								0%	Pos
	Lung metastases (13)	1.85 (1.22)	No	0.11	0.74	6.2	0.08	2.31	-	-
25	Resected primary tumor								5%	Pos
	Neck metastases (7)	4.63 (1.29)	No	1.16	0.54	11.6	0.22	4.63	-	-
	Lung metastasis (1)	4.50	Yes	1.13	0.53	11.3	0.21	4.50	-	-
	Pericardial metastasis (1)	3.20	No	0.80	0.38	8.0	0.15	3.20	-	-
	Distant lymph nodes (4)	3.83 (1.16)	Yes	0.96	0.45	9.6	0.18	3.83	-	-
	Subcutaneous metastasis (1)	2.90	Yes	0.73	0.34	7.3	0.13	2.90	-	-

## Abbreviations

^18^F: Fluor-18
^68^Ga: Gallium-68
^177^Lu: Lutetium-177
^225^Ac: Actinium-225
ACC: adenoid cystic carcinoma
ADT: androgen deprivation therapy
AR: androgen receptor
CAP: cyclophosphamide plus doxorubicin plus cisplatin
CRPC: castration resistant prostate cancer
CT: computed tomography
FFPE: formalin-fixed, paraffin-embedded
IHC: immunohistochemistry
mCRPC: metastatic castration resistant prostate cancer
MRI: magnetic resonance imaging
PET: positron-emission tomography
PSMA: prostate-specific membrane antigen
R/M: recurrent and/or metastatic
SD: standard deviation
SDC: salivary duct carcinoma
SGC: salivary gland cancer
SUV: standardized uptake values
ULN: upper limit of normal
