# Supplementary material for: 68Ga-PSMA-HBED-CC PET/CT imaging for adenoid cystic carcinoma and salivary duct carcinoma: a phase 2 imaging study
Source: Theranostics. 2020 Jan 12;10(5):2273–83. doi: 10.7150/thno.38501 (PMC7019174; doi:10.7150/thno.38501)

Supplementary table 1: Patient characteristics of adenoid cystic carcinoma patients (No. 1-15) and salivary duct carcinoma (SDC) patients (No 16-25). All SDCs were androgen-receptor-positive. mo.: months. T: local recurrence of primary tumor. N: Regional lymph node metastases. M: distant metastases. * patient no. 4 had distant metastases in the pleura, lung, pericard, myocard, subcutaneous tissue, and bone.

**Adenoid cystic carcinoma patients**										
**No.**	**Gender**	**Age**	**Time from diagnosis (mo.)**	**Time from recurrence (mo.)**	**Location primary tumor**	**ACC subtype**	**Primary tumor resection**	**Postoperative radiotherapy**	**Location of recurrence**	**Current treatment**
1	Male	62	88	64	Minor salivary glands	Solid and cribriform	Yes	Yes	N,M (lung)	Watchful waiting
2	Female	68	251	14	Bartholin gland	Cribriform	Yes	Yes	M (lung)	Watchful waiting
3	Male	58	89	72	Trachea	Solid	Yes	Yes	M (lung)	Vorinostat 400 mg OD
4	Male	56	77	58	Bronchus	Cribriform	Yes	Yes	T, M (*)	None: baseline scan before start of treatment
5	Female	64	35	3	Submandibular gland	Cribriform	Yes	Yes	M (lung)	Watchful waiting
6	Male	44	135	75	Minor salivary glands	Solid and cribriform	Yes	Yes	M (bone)	Watchful waiting
7	Female	54	59	27	Parotid gland	Tubular	Yes	Yes	T, N	Watchful waiting
8	Male	76	93	54	Minor salivary glands	Cribriform	No	Yes	M (lung)	Watchful waiting
9	Male	53	90	52	Minor salivary glands	Cribriform	Yes	Yes	M (pleura, bone)	Watchful waiting
10	Male	59	36	28	Parotid gland	Cribriform	Yes	No	M (lung)	Watchful waiting
11	Male	45	49	17	Parotid gland	Cribriform	Yes	Yes	T, M (kidney)	Watchful waiting
12	Female	64	170	3	Submandibular gland	Cribriform	Yes	Yes	M (lung)	Watchful waiting
13	Male	55	54	46	Parotid gland	Cribriform	Yes	Yes	M (lung, liver)	cyclophosphamide 500 mg/m^2 plus doxorubicin 50 mg/m^2 plus cisplatin 50 mg/m^2
14	Male	58	176	16	Minor salivary glands	Solid	Yes	No	M (lung, bone)	Cabozantinib 40 mg OD
15	Male	64	6	6	Sublingual gland	Cribriform	Yes	No	M (lung)	Watchful waiting
**Salivary duct carcinoma patients**										
**No.**	**Gender**	**Age**	**Time from diagnosis (mo.)**	**Time from recurrence (mo.)**	**Location primary tumor**	**HER2 status**	**Primary tumor resection**	**Postoperative radiotherapy**	**Location of recurrence**	**Current treatment**
16	Male	75	66	13	Parotid gland	Positive	Yes	Yes	N, M (bone)	Goserelin 10.8 mg per 3 months plus bicalutamide 50 mg OD
17	Male	55	16	1	Submandibular gland	Negative	Yes	Yes	N, M (lung, distant lymph nodes)	None: baseline scan before start of treatment
18	Male	79	8	1	Parotid gland	Negative	Yes	Yes	T, M (lung, bone, distant lymph nodes)	None: baseline scan before start of treatment
19	Male	66	41	35	Parotid gland	Positive	Yes	Yes	N, M (lung, distant lymph nodes)	Goserelin 10.8 mg per 3 mo. plus bicalutamide 50 mg OD

20	Male	70	74	67	Parotid gland	Negative	Yes	Yes	T	Goserelin 10.8 mg per 3 mo. plus bicalutamide 50 mg OD
21	Male	58	9	8	Parotid gland	Positive	Yes	No	T, M (brain, bone)	None: baseline scan before start of treatment
22	Male	75	45	22	Parotid gland	Positive	Yes	Yes	M (liver, bone, subcutaneous, distant lymph nodes)	Trastuzumab 6 mg/kg plus pertuzumab 420 mg fixed dose
23	Male	66	42	23	Parotid gland	Negative	Yes	Yes	M (bone)	Goserelin 10.8 mg per 3 mo. plus bicalutamide 50 mg OD
24	Male	69	21	21	Parotid gland	Positive	No	No	M (lung)	Trastuzumab-emtansin 3.6 mg/kg
25	Male	73	61	47	Parotid gland	Unknown	Yes	Yes	N, M (lung, pericardial, subcutaneous, distant lymph nodes)	Bicalutamide 150 mg OD

Supplementary table 2: Background maximum and mean standardized uptake value's (SUV_{max} and SUV_{mean}) in adenoid cystic carcinoma (ACC) and salivary duct carcinoma (SDC) patients. SUV_{mean} was measured with a 2.4 cm diameter of the region of interest. SD: standard deviation. * SUV_{mean} could not be calculated reliably in the lacrimal gland because measurement with a ROI of 2.4 cm exceeds the size of the gland, and a smaller ROI results in in a partial volume effect.

	ACC		SDC	
	**Mean SUV_{max} ± SD (range)**	**Mean SUV_{mean} ± SD (range)**	**Mean SUV_{max} ± SD (range)**	**Mean SUV_{mean} ± SD (range)**
Lacrimal gland	23.87 ± 9.54 (14.9 – 49.5)	*	24.06 ± 8.20 (14.4 – 42.5)	*
Parotid gland	26.17 ± 10.45 (14.9 – 56.5)	16.19 ± 6.18 (7.8 – 33.0)	23.15 ± 6.68 (7.3 – 32.1)	14.18 ± 3.99 (4.0 – 17.2)
Submandibular gland	26.49 ± 11.93 (9.8 – 55.5)	11.74 ± 7.26 (3.2 – 29.6)	23.08 ± 7.00 (12.3 – 35.5)	9.02 ± 4.04 (3.3 – 16.7)
Sublingual gland	16.93 ± 5.69 (4.3 – 22.6)	5.91 ± 2.92 (2.1 – 11.0)	17.81 ± 6.70 (8.5 – 30.5)	6.30 ± 3.78 (2.5 – 12.7)
Lung	1.38 ± 0.36 (0.8 – 2.0)	0.64 ± 0.19 (0.3 – 1.1)	1.07 ± 0.29 (0.5 – 1.5)	0.52 ± 0.13 (0.3 – 0.7)
Mediastinal bloodpool	2.16 ± 0.52 (1.5 – 3.3)	1.14 ± 0.32 (0.8 – 2.1)	2.06 ± 0.48 (1.2 – 2.7)	1.14 ± 0.21 (0.8 – 1.5)
Liver	6.31 ± 1.24 (4.8 – 9.4)	4.00 ± 0.90 (2.5 – 6.2)	6.47 ± 2.73 (3.2 – 12.4)	4.33 ± 2.02 (2.0 – 8.5)
Duodenum	23.02 ± 7.54 (12.3 – 37.2)	10.73 ± 3.95 (5.0 – 19.2)	18.66 ± 4.67 (11.5 – 25.6)	8.96 ± 1.47 (6.6 – 11.1)
Spleen	14.84 ± 6.22 (6.1 – 27.2)	11.09 ± 5.15 (4.2 – 22.1)	10.19 ± 7.36 (5.2 – 29.7)	7.14 ± 4.20 (3.7 – 17.5)
Kidney	68.04 ± 25.83 (42.3 – 131.6)	37.04 ± 14.56 (22.1 – 77.5)	57.41 ± 23.31 (18.3 – 102.4)	32.73 ± 14.30 (11.7 – 63.1)
Bladder	19.12 ± 7.36 (6.5 – 31.7)	13.35 ± 4.91 (4.4 – 20.6)	15.55 ± 5.32 (10.0 – 25.5)	12.09 ± 4.43 (7.7 – 21.2)
Gluteus muscle	0.95 ± 0.24 (0.5 – 1.4)	0.39 ± 0.12 (0.2 – 0.7)	0.88 ± 0.12 (0.6 – 1.0)	0.36 ± 0.07 (0.3 – 0.5)

Supplementary figure 1: Scatter plot of the time from recurrence to PSMA-PET scan and maximum standardized uptake value's (SUVmax) in recurrent and metastatic lesions of ACC patients (**A**) and SDC patients (**B**). Note that patients with x disease site are represented in the figures x times.

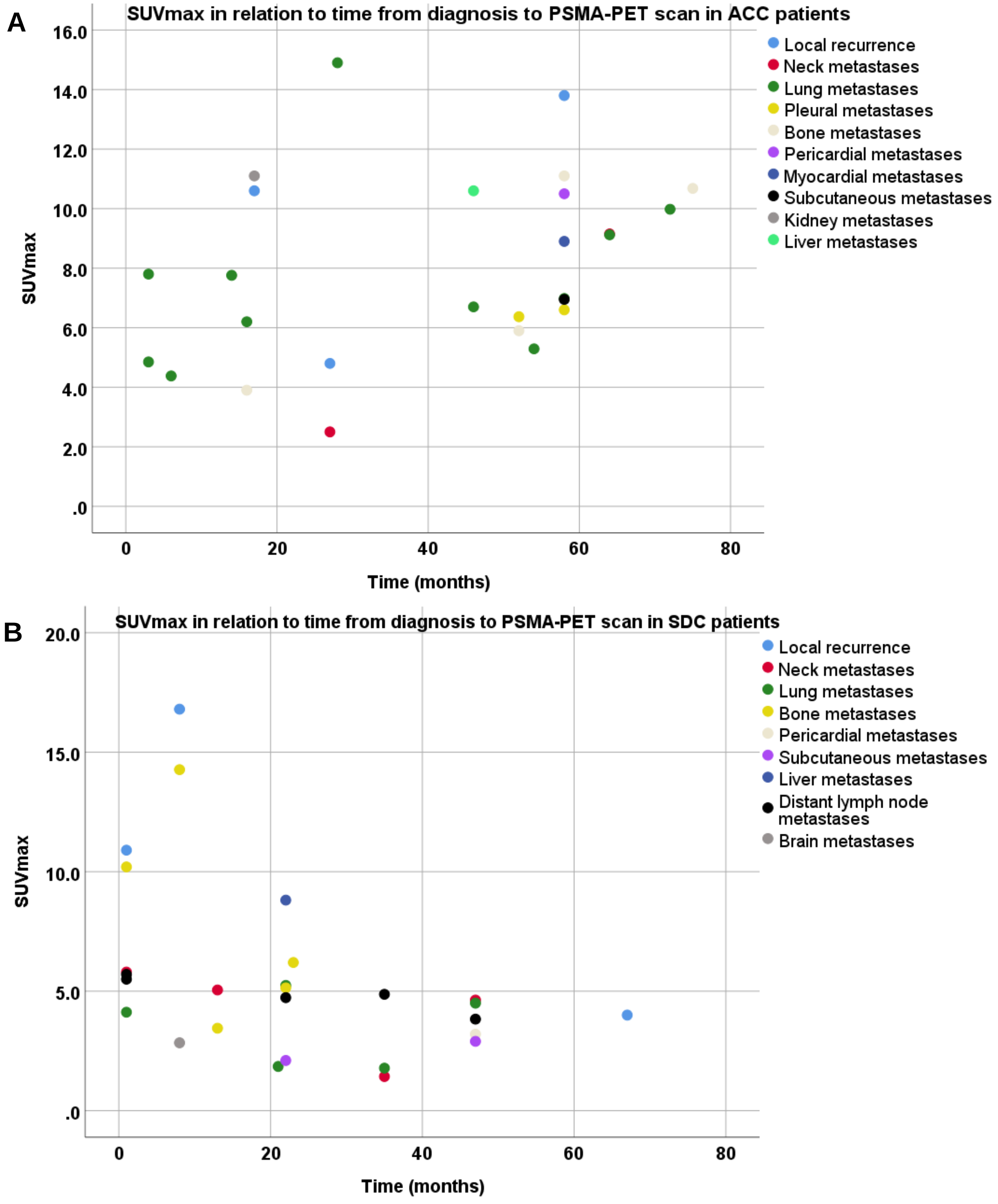

Supplementary figure 2: Scatter plot of the percentage of prostate-specific membrane antigen (PSMA)-positive tumor cells in the resected primary tumor and mean SUV_{max} in recurrent/metastastic disease sites in the same patient, shown for adenoid cystic carcinoma (ACC) patients (**A**) and salivary duct carcinoma (SDC) patients (**B**). A scatter plot of the percentage of PSMA-positive tumor cells in biopsied recurrent and/or metastatic lesions and mean SUV_{max} in the same disease site in the same patient, is shown for ACC patients (**C**).

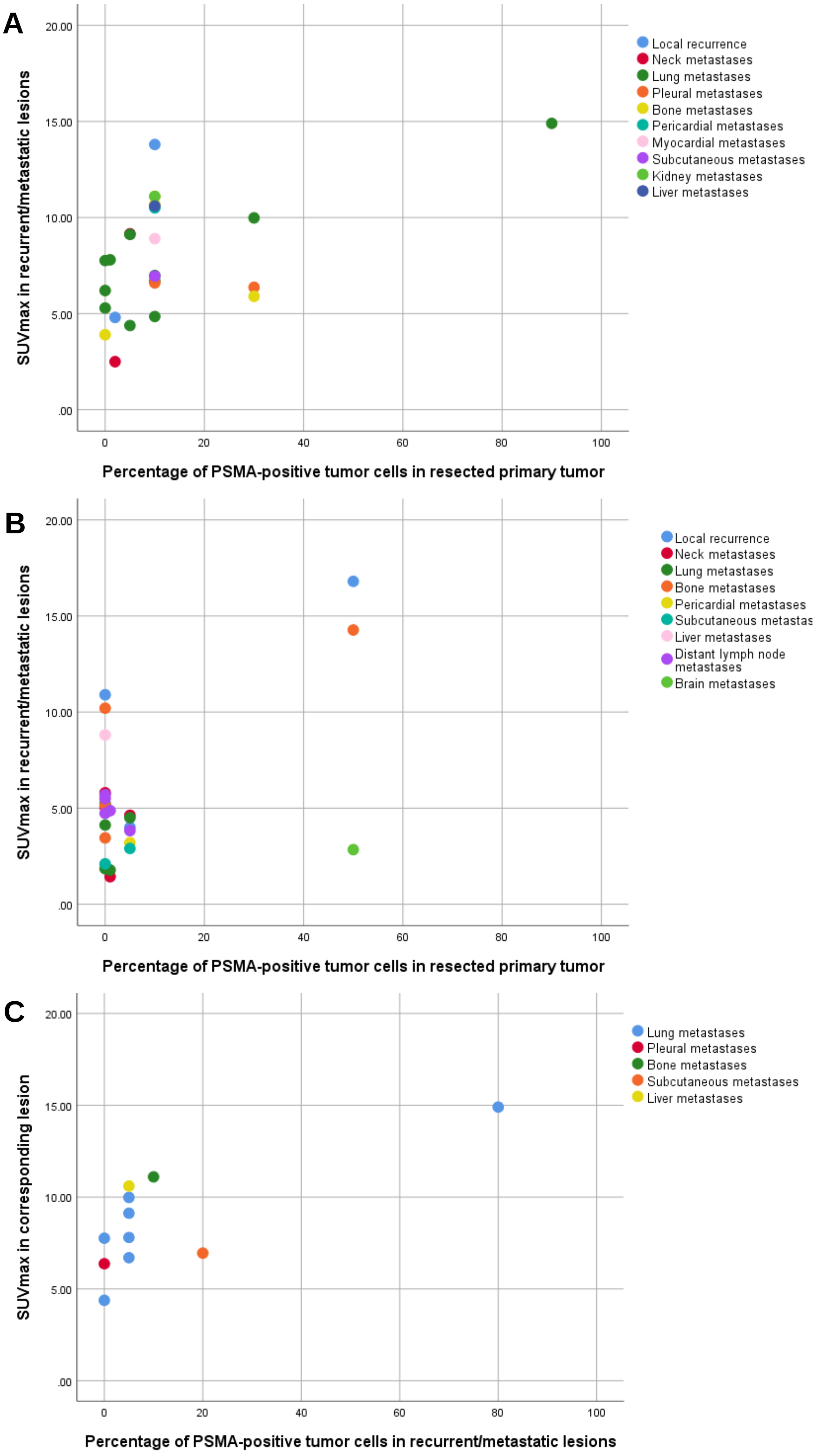

Supplement: Supplementary file 1 — Supplementary figures and tables. [file thnov10p2273s1.pdf]
